# Artificial Intelligence in Medical Applications

**DOI:** 10.1155/2018/4827875

**Published:** 2018-07-15

**Authors:** Yung-Kuan Chan, Yung-Fu Chen, Tuan Pham, Weide Chang, Ming-Yuan Hsieh

**Affiliations:** ^1^Department of Management Information Systems, National Chung Hsing University, No. 250, Kuokuang Rd., Taichung 402, Taiwan; ^2^Department of Dental Technology and Materials Science, Central Taiwan University of Science and Technology, No. 666, Buzih Road, Beitun District, Taichung 40601, Taiwan; ^3^Department of Biomedical Engineering, Linköping University, 462 Entrance 71, Floor 12, Room 403, Campus US, Linköping, Sweden; ^4^Department of Computer Science, California State University, 6000 J Street, Sacramento, CA 95819-6021, USA; ^5^Department of International Business, National Taichung University of Education, No. 140, Minsheng Rd., West District, Taichung 40306, Taiwan

Medical artificial intelligence (medical AI) mainly uses computer techniques to perform clinical diagnoses and suggest treatments. AI has the capability of detecting meaningful relationships in a dataset and has been widely used in many clinical situations to diagnose, treat, and predict the results. In the research and studies of medical AI, we primarily focus on the viability and feasibility to incorporate various computer AI techniques in medical information modeling and clinical procedure deployments. The state-of-the-art AI methodologies have shown great capabilities and capacities in recognition of meaningful data patterns and thus been widely experimented as tools for clinical trials, especially, to aid the decision making in each phase for diagnoses and subsequent treatments, as well as prognoses and projections.

The main focus of this special issue is on the proposal of techniques for medical artificial intelligence, expert systems, data mining, machine learning, and image processing which could be built on top of them. This special issue summarizes the most recent developments in the field, with a special emphasis given to the improvements and results obtained within the last several years. With highly measurable improvements, the MAI issue demonstrates the great potential and promises of applying AI techniques to pragmatic clinical procedures and analytical medical informatics, especially in the following areas:

- Artificial Intelligence Techniques in Medicine
- Data Mining and Knowledge Discovery in Medicine
- Medical Expert Systems
- Machine Learning-Based Medical Systems
- Medical Signal and Image Processing Techniques

Thirty-three papers were submitted for this special issue. Our distinguished reviewers from respective research fields narrowed the field to eleven papers which were finally accepted. The following is a short summary of the findings of each of these papers.

Avila-Garcia et al. demonstrated the use of a neural network-based multiscale Gaussian matching filter for detection and segmentation on coronary angiogram X-ray images and thus effectively enhanced the results of image classification.

Cui et al. proposed a cascaded neural network composed of a Tumor Localization Network to localize the brain tumor from slices of MRI images and an Intra-Tumor Classification Network to label tumor regions. With advanced technologies used to train and optimize the cascaded neural network, it attained better accuracy and computation efficiency over other neural network-based methods.

Fu et al. published their results of using a Convolutional Neural Network (CNN) to recognize strabismus. The CNN was trained from the data collected by an eye-movement tracker, including Gaze Deviation (GaDe) image data from both subjects of normal and strabismic visions. After trained with a large number of GaDe images, their CNN performed successfully for strabismus recognition.

Chan et al. deployed a support vector machine to effectively detect common pneumothorax by using the local binary patterns obtained through a multiscale intensity texture analysis on the chest X-ray images.

Chen et al. designed a clinical decision support system to predict fractures in hip bones and vertebrates caused by medications for treatments of chronic respiratory diseases. The system was designed with an integrated genetic algorithm and support vector machine through training with balanced datasets obtained from random and cluster-based undersampling methods, as well as tested with imbalanced datasets.

Barnes et al. proposed a data-driven predictive modeling framework for decision support systems, based on evolutionary computation techniques to optimize multilevel data. The framework could be built from open-source software and flexible to include other evolutionary algorithms.

Kim et al. proposed a Deep Belief Network and Dempster–Shafer- (DBN-DS-) based multiclassifier for the pathologic prediction of prostate cancer, tested with data from thousands of patients, and obtained high accuracy (81.27%) against the Partin table (64.14%).

Kaur et al. examined in detail the performances of hybrid computing models used to predict blood pressures (BP). Each hybrid model was formed with principal component analysis (PCA) and one of the following: Forward Stepwise Regression (FSWR), Artificial Neural Network (ANN), Adaptive Neuro-Fuzzy Inference System (ANFIS), and Least Square-Support Vector Machine (LS-SVM). The BP predicted was associated with reactivity to crossed legs among normotensive and hypertensive participants. The LS-SVM model achieved significant improvements.

Yang experimented with 3D Shape-Weighted Level Set Method (3D-SLSM) to carry out precise segmentation of tumors from 3D medical images. The segmentation results of most 3D segmentation algorithms were affected largely by errors and noise; however, 3D-SLSM added 3D shape-weighted values in each iterative process according to the volume changes. The accuracy of 3D-SLSM was tested with MRI and computer-generated images and had the highest accuracy and lowest false-positive rate when compared with the standard tumor model.

Lin et al. researched on effects of outlier removals in medical datasets, that is, whether performing instance selection to filter out noisy data from a given training medical dataset has a positive impact on the final imputation results. By comparing the classification performance obtained through the processes combining instance selection and imputation and the baseline imputation process, three types of medical datasets are used: categorical, numerical, and mixed types. Three different instance selection methods and three model-based imputation algorithms were compared. The experimental results showed that performing instance selection first mostly improves the imputation result over the types of medical data. Further, the negative impact was to consider instance selection before imputation when the dataset contains lower dimensionalities and numbers of classes. However, for numerical datasets, the combined instance selection and imputation process would perform better than the baseline imputation process for most datasets with different missing rates. They also concluded that for mixed datasets, the instance selection effect was between that for categorical and numerical datasets, which meant that combining instance selection and imputation could be a better choice when algorithms were carefully chosen.

Chen devised a personalized antidiabetic medication recommendation system that tailored HbA1c levels to mitigate medical differences for the selection of antidiabetic medications. By combining fuzzy logic and ontology reasoning, the system could promote a “patient-centric diabetes therapy” besides aiding clinical diagnosis and assist family practitioners in prescribing medications.

*Yung-Kuan Chan* *Yung-Fu Chen* *Tuan Pham* *Weide Chang* *Ming-Yuan Hsieh*

